# Rifampicin‐Induced Lung Injury in *Mycobacterium shinjukuense* Infection: A Case Report

**DOI:** 10.1002/rcr2.70208

**Published:** 2025-05-12

**Authors:** Yoshimasa Hachisu, Masako Hanawa, Yuki Hosino, Shogo Uno, Yuji Onuki, Kazuma Ezawa, Takeo Horie

**Affiliations:** ^1^ Department of Respiratory Medicine Maebashi Red Cross Hospital Maebashi‐city Japan

**Keywords:** drug‐induced lung injury, *Mycobacterium shinjukuense*, NTM‐PD, organising pneumonia, rifampicin

## Abstract

A 70‐year‐old woman with sputum and cough was diagnosed with nontuberculous mycobacterial pulmonary disease based on multiple cultures of *Mycobacterium shinjukuense* and computed tomography (CT) findings of bilateral nodular shadows and bronchiectasis. Treatment was initiated with ethambutol and azithromycin, and rifampicin was added 1 month later. Three weeks after rifampicin therapy, she developed fever and fatigue, and CT revealed bilateral non‐segmental infiltrates and a reversed halo sign indicative of organising pneumonia. Rifampicin was discontinued due to suspected rifampicin‐induced lung injury, and a subsequent bronchial lung biopsy confirmed organising pneumonia. The patient's fever resolved with discontinuation of rifampicin, and chest imaging showed resolution of the infiltrates. Continuation with ethambutol and azithromycin did not result in fever or new shadows. We need to consider the possibility of drug‐induced lung injury when new shadows appear during nontuberculous mycobacterial treatment regimens.

## Graphical abstract

We report a case of drug‐induced lung injury caused by rifampicin during the treatment of *M. shinjukuense* infection, with a review of the literature.

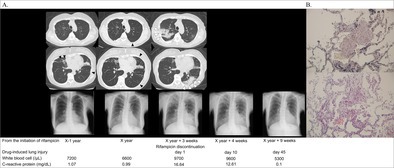

## 1 Introduction

Nontuberculous mycobacterial infectious pulmonary disease (NTM‐PD) is an increasing lung infection worldwide, and *Mycobacterium shinjukuense* is a relatively infrequent species identified in recent years [1, 2]. Rifampicin (RFP)‐containing regimens are often used to treat nontuberculous mycobacterial infections, whereas RFP, clarithromycin (CAM), and ethambutol (EB) are often used in *M. shinjukuense* [3].

Although RFP is known to cause lung injury as a side effect of pulmonary tuberculosis [4], there are no reports of lung injury in patients with NTM‐PD as far as we have examined.

Here, we report a case of drug‐induced lung injury caused by RFP during the treatment of *M. shinjukuense* infection, with a review of the literature.

## 2 Case Report

A 70‐year‐old woman presented to our hospital complaining of sputum production and a cough. The patient was treated for malignant lymphoma in year X‐13 and achieved complete remission. There were no other immunosuppressive conditions, such as additional malignancies or HIV infection. Computed tomography (CT) revealed nodular shadows in the upper lobes of both lungs and the middle lobe of the right lung as well as bronchiectasis in the middle lobe of the right lung and the lingular segment of the left lung. *M. shinjukuense* was cultured from her sputum once in year X‐3, and once from three samples within a two‐week period in year X, leading to a diagnosis of NTM‐PD.

**FIGURE 1:**
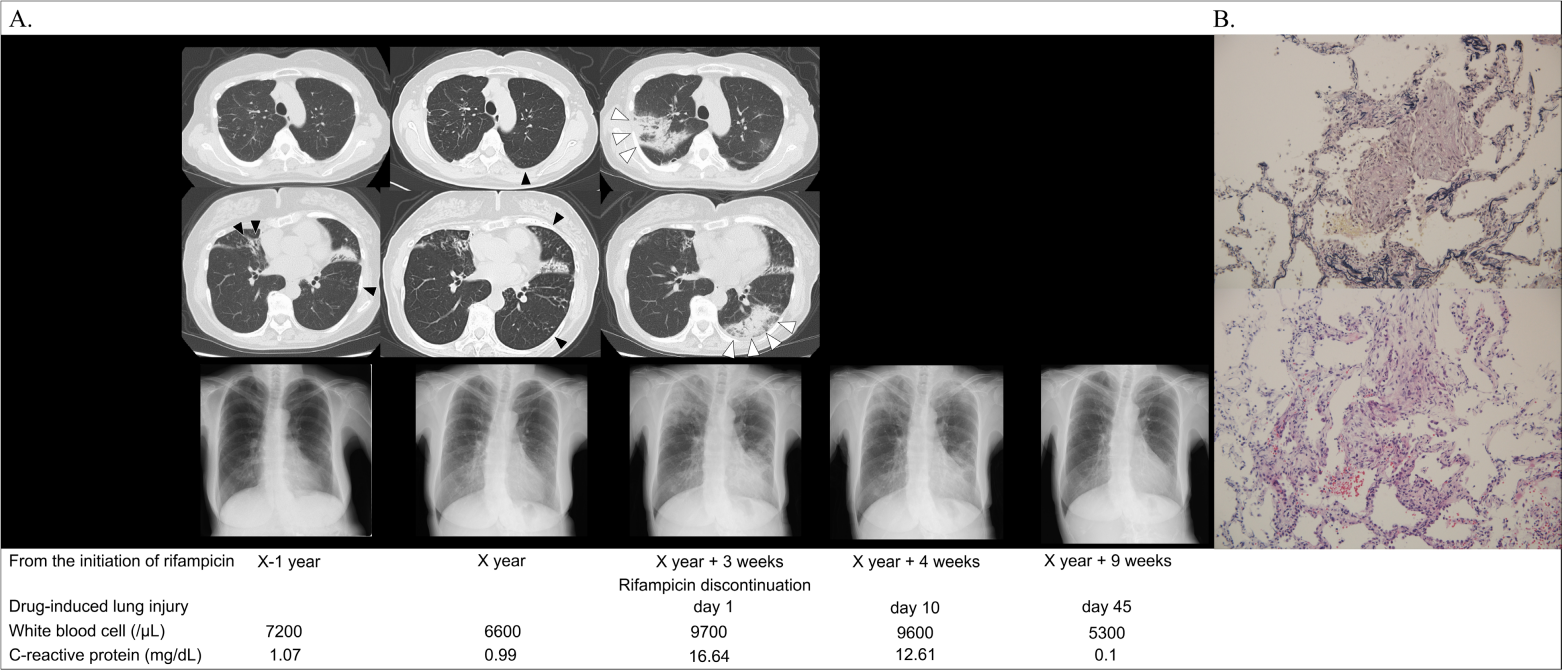
Progression of Chest X‐ray and CT scan image. (A) The patient is diagnosed with *Mycobacterium. shinjukuense* infection in *X* − 1 year and starts treatment in *X* year. Initially, nodular shadows and bronchiectasis are observed in both lungs (black arrowhead), with slow progression over time. *X* year plus 3 weeks later, she has a fever, and a CT scan shows new infiltrate shadows in both lungs, partly with a reversed halo sign (white arrowhead). Rifampicin is discontinued, the fever gradually resolves, and the lung shadows disappear clearly by *X* years plus 9 weeks. (B) A transbronchial lung biopsy is performed from S10 of the lower lobe of the left lung. The pathology slide shows a 200× EVG and HE‐stained specimen. Fibroblast proliferation is consistent with organising pneumonia.

Susceptibility testing was performed using BrothMIC NTM (Kyokuto Pharmaceutical Industrial), and the minimum inhibitory concentrations were as follows: EB, 0.5; RFP, ≤ 0.03; and CAM, ≤ 0.03, indicating good susceptibility. Haematological tests showed a white blood cell count of 6600/μL composed of 60.5% neutrophils, 25.8% lymphocytes, 2.9% eosinophils, and 9.6% monocytes, AST levels of 20 U/L, ALT levels of 11 U/L, and CRP level of 0.99 mg/dL. There was no history of drug‐induced liver injury, but the patient was highly concerned about gastrointestinal side effects; she began treatment with two drugs, EB and azithromycin (AZM), and RFP was added 1 month later. Three weeks after initiating RFP, the patient developed fever and fatigue. Blood tests showed elevated C‐reactive protein levels (Table 1), and computed tomography (CT) revealed non‐segmental infiltrates in both the upper and lower lobes of the lungs, with a reversed halo sign observed in the lower lobe of the left lung (Figure 1). The initially observed bronchiectasis in the middle and lingual lobes remained unchanged, while the nodular shadows became difficult to discern due to modification by the infiltrates. The reversed halo sign raised suspicion for organising pneumonia, in addition to infection, granuloma, and tumour. Blood tests revealed no findings suggestive of secondary organising pneumonia. Drug‐induced lung injury due to RFP was suspected, as only three drugs had been initiated within the past 3 months, and there were no reports of drug‐induced lung injury associated with EB or AZM. RFP was discontinued, and levofloxacin was administered for five days to rule out bacterial infection; however, her fever persisted. Bronchoscopy was performed for further investigation. Bronchoalveolar lavage was conducted in the left lung B1 + 2 segment, and a transbronchial lung biopsy was taken from the left lung B10 segment, which showed infiltrates on CT. Bronchoalveolar lavage fluid revealed no increase in lymphocytes, but tissue biopsy with transbronchial lung biopsy showed fibrous thickening of the alveolar septa and proliferation of fibroblasts, consistent with organising pneumonia. No cultures of routine or acid‐fast bacteria were observed in bronchial washings. Considering the possibility of drug‐induced lung injury due to a delayed allergic reaction, a drug‐induced lymphocyte stimulation test (DLST) was performed as an adjunctive diagnostic tool to evaluate the potential for re‐administration, which gave a negative result for RFP.

**TABLE 1:** Laboratory data at the onset of drug‐induced lung injury.

Haematological data					
White blood cells	9700/μL	Total protein	6.8 g/dL	BNP	24.0 pg/mL
Neutrophils	79.7%	Albumin	2.4 g/dL	KL‐6	256 U/mL
Lymphocytes	9.0%	Total bilirubin	0.7 mg/dL	Rheumatoid factor	11 IU/mL
Eosinophils	1.9	AST	13 U/L	SP‐A	71.0 ng/mL
Basophils	0.4%	ALT	11 U/L	SP‐D	225 ng/mL
Monocytes	9.0%	ALP	108 U/L	Anti‐Nuclear‐Antibody	< 40
Red blood cells	401 × 10^−4^/μL	γ‐GTP	44 U/L	Anti‐CCP‐Antibody	0.6 U/mL
Haemoglobin	11.7 g/dL	LDH	162 U/L	Anti‐ARS‐Antibody	5.8 U/mL
Haematocrit	36.7%	Total cholesterol	188 mg/dL	PR3‐ANCA	1.0 U/mL
Platelets	42.2 × 10^−4^/μL	BUN	15 mg/dL	MPO‐ANCA	4.3 U/mL
		Creatinine	0.57 mg/dL		
PT%	74%	Sodium	137 mEq/L		
PT‐INR	1.17	Potassium	4.9 mEq/L		
APTT	31.8 s	Chloride	101 mEq/L		
Fibrinogen	1011 mg/dL	Calcium	8.7 mg/dL		
D‐dimer	1.9 μg/mL	C‐reactive protein	16.64 mg/dL		
		Glucose	233 mg/dL		
		HbA1c	6.4%		
BALF				Drug‐induced lymphocyte stimulation test	
Neutrophils	11.0%	CD4/8 ratio	1.02	Rifampicin	S.I. 144% negative
Lymphocytes	15.0%	Recovery rate	77/150 mL		
Eosinophils	2.0%				
Basophils	0.0%	Routine culture	Negative		
Monocytes	72.0%	Mycobacterium culture	Negative		
		Cytology	Negative		

Abbreviations: ARS: Aminoacyl tRNA synthetase, BALF: bronchoalveolar lavage fluid, CCP: cyclic citrullinated peptide, KL‐6: Krebs von den lungen‐6, MPO‐ANCA: myeloperoxidase anti‐neutrophil cytoplasmic antibody, PR3‐ANCA: proteinase 3 anti‐neutrophil cytoplasmic antibody, SP‐A: surfactant protein‐A, SP‐D: surfactant protein‐D.

No respiratory failure was observed; therefore, glucocorticoids were not administered and the patient was treated with drug discontinuation only. She experienced fever for nine days after stopping the drug, which subsided on the 10th day and did not recur thereafter. Chest radiography on the 10th day showed persistent infiltrates; however, by the 15th day, the infiltrates in the lower lung fields of both lungs had regressed. Six weeks after fever onset, the infiltrates had nearly disappeared. At 4 months post‐fever, EB and AZM were still administered, with no re‐exacerbation of fever or shadow.

## 3 Discussion

RFP is a drug used to treat NTM‐PD and can cause several side effects, including thrombocytopenia and abnormal liver function tests [5]. Drug‐induced lung injury due to RFP is known to occur in patients treated for pulmonary tuberculosis with symptoms including fever, cough, and dyspnea [4]. To the best of our knowledge, there have been no reports of rifampicin‐induced lung injury in patients with NTM‐PD. Furthermore, *M. shinjukuense* is a rare species, and no side effects related to its treatment have been previously reported.

RFP‐induced lung injury may result from an immunological response [6]. It has also been reported to be associated with RFP‐induced liver injury and may be relevant to lung injury [7]. Cases of lung injury show the appearance of bilateral lung infiltrate shadows, increased lymphocyte counts in the bronchoalveolar lavage fluid, and a positive DLST for RFP. However, the sensitivity of DLST is low and has been reported to be 13.6% for RFP in tuberculosis [8]. The pathogenesis of drug‐induced lung injury is complex. In addition to T cell‐dependent immune responses, direct cytotoxicity by drugs is also known to play a role. DLST evaluates T cell proliferative responses; however, careful interpretation of the results is necessary. Some patients improved with drug discontinuation alone, whereas others required corticosteroid administration.

The 2020 ATS/ERS/ESCMID/IDSA guidelines recommend diagnosing NTM‐PD when the same species is identified two or more times in different sputum specimens, and bacterial susceptibility testing is recommended when the diagnosis is confirmed [9]. For *Mycobacterium avium* complex disease, regimens including AZM are preferred over CAM due to better tolerability and fewer drug interactions. *M. shinjukuense* is a slow‐growing, non‐tuberculous mycobacterium that was first identified in Japan in 2004 [2] and is often treated empirically with three drugs: RFP, EB, and CAM, or isoniazid, RFP, and EB [3]. In this case, based on previous reports and guideline recommendations, the patient was treated with a three‐drug regimen comprising RFP, EB, and AZM.

In the present case, no new radiographic abnormalities were observed following the administration of EB or AZM; however, the addition of RFP resulted in diffuse ground‐glass opacities and infiltrates. Although the DLST for RFP was negative, the serological and radiographic conditions of the patient improved only after discontinuation of RFP. As EB and AZM could be continued without recurrence, the patient was diagnosed with drug‐induced lung injury. In drug‐induced lung injury, the reproducibility of symptoms with re‐administration strongly supports the diagnosis; however, this could not be performed in this case.

There have been several reports of RFP‐induced lung injury in pulmonary TB, which differs from NTM‐PD in that many drug regimens are used, the bacterial growth rate is rapid, and biofilm formation is not observed. However, it is unclear whether these factors contribute to the incidence of lung injury. Because pulmonary TB requires early initiation of treatment, there have been many reports of adverse drug reactions; however, because the need for treatment of NTM‐PD has been underestimated, there have been few cases of treatment, and there may be an underreporting bias in the number of reports.

The number of patients with NTM‐PD is increasing worldwide, and those treated with RFP‐containing regimens for NTM‐PD are also increasing. The possibility of drug‐induced lung injury due to RFP should be considered when new shadows appear during the treatment of patients with NTM‐PD.

## Author Contributions

Y.H. managed patients, collected data and wrote the original manuscript. All authors revised, reviewed and approved the final version of the manuscript.

## Ethics Statement

The authors declare that written informed consent was obtained for the publication of this manuscript and accompanying images using the consent form provided by the Journal.

## Conflicts of Interest

The authors declare no conflicts of interest.

## Data Availability

The data that support the findings of this study are available from the corresponding author upon reasonable request.
